# A Retrospective Histological Study on Palatal and Gingival Mucosa Changes during a Rapid Palatal Expansion Procedure

**DOI:** 10.3390/biomedicines11123246

**Published:** 2023-12-07

**Authors:** Eugen Bud, Alexandru Vlasa, Mariana Pacurar, Adrian Matei, Anamaria Bud, Andreea-Raluca Szoke, Giuseppe Minervini

**Affiliations:** 1 Department of Orthodontics and Dental-Facial Orthopedics, Faculty of Dental Medicine, George Emil Palade University of Medicine and Pharmacy, Science and Technology, 540139 Târgu-Mureș, Romania; eugen.bud@umfst.ro (E.B.); mariana.pacurar@umfst.ro (M.P.); 2 Department of Periodontology and Oral-Dental Diagnosis, Faculty of Dental Medicine, George Emil Palade University of Medicine and Pharmacy, Science and Technology, 540139 Târgu-Mureș, Romania; 3 Independent Researcher, 540139 Târgu-Mureș, Romania; 4 Department of Pedodontics, Faculty of Dental Medicine, George Emil Palade University of Medicine and Pharmacy, Science and Technology, 540139 Târgu-Mureș, Romania; 5 Department of Physiopathology, Faculty of General Medicine, George Emil Palade University of Medicine and Pharmacy, Science and Technology, 540139 Târgu-Mureș, Romania; andreea-raluca.szoke@umfst.ro; 6 Multidisciplinary Department of Medical-Surgical and Dental Specialties, University of Campania, Luigi Vanvitelli, 80138 Naples, Italy; giuseppe.minervini@unicampania.it

**Keywords:** rapid palatal expansion, mini-implant-assisted rapid palatal expander, MARPE, gingival overgrowth, hyperplasia

## Abstract

The most common inflammatory reactions in the oral mucosa are found at the gingival level. The treatment of these inflammations requires, first of all, the removal of the causative factor; often, this maneuver is sufficient. The aim of this retrospective study was to evaluate clinical and histopathological changes that occur in terms of gingival and palatal mucosa enlargement during palatal expansion treatment and their evolution during treatment. Twenty-five (*n* = 25) research participants, aged between thirteen and twenty-six years old, were examined in this retrospective study. At the end of the treatment, fragments of tissue from the affected level were obtained via incisional biopsy and sent to the histopathology laboratory for a specialized examination. The changes identified were specific to mechanical traumatic injuries, thus excluding hyperplasia from other etiologies (infectious, tumoral, or non-mechanical traumatic). The examined fragments showed hyperplasia. The histopathological examination revealed the mechanical character of the lesion, strengthening the causal relationship between the insertion of the expander and the occurrence of hyperplasia of the palatal mucosa. The type of palatal expander influenced the degree of inflammation, with the severity of hyperplasia being more pronounced in the case of mini-implant-anchored rapid palatal expander (MARPE) usage than in the case of tooth-borne rapid palatal expander (RPE) usage. The analysis of the distance between the expander and the palatal mucosa did not provide conclusive results; the incidence and severity of the reaction were variable in patients with the same distance between the expander and the palatal or gingival mucosa.

## 1. Introduction

The mucosa of the oral cavity is in direct contact with the environment and is frequently subjected to multiple sources of damage. Its reduced thickness is a factor that makes it even more susceptible to damage, especially that of traumatic origin (mechanical, physical, or chemical) [1]. The most common inflammatory reactions in the oral mucosa are found at the gingival level. According to etiology and pathological changes, they are classified into inflammatory hyperplasia (chronic or acute), drug-induced hyperplasia, hyperplasia associated with systemic diseases, gingival tumors, and pseudo-hyperplasia [2]. In addition to these forms, hyperplasia of the mucosa can occur at the lingual or palatal level. Regardless of the location, making the differential diagnosis is essential for developing a correct treatment plan [3]. Patients presenting to dental clinics may present with inflammation of the oral mucosa that does not respond to improved oral hygiene or periodontal treatment. This fact indicates the presence of a specific etiological factor [4]. The treatment of these inflammations requires, first of all, the removal of the causative factor; often, this maneuver is sufficient [5]. In cases of severe inflammation of traumatic origin, in addition to removing the cause, surgical excision of the tissue fragment is recommended, and in inflammations of toxic origin, specific drug treatment is indicated [6].

Rapid palatal expansion (RPE) is a treatment method used to correct the reduced size of the jaw. It involves separating the medial palatine suture to widen the maxillary basal bone in young children, adolescents, and adults. The procedure has been used for more than a century in orthodontic therapy, and its effects on dentition and the craniofacial skeleton have been studied and documented by various authors [7,8,9].

The application of the palatal expander is an invasive maneuver and, as such, may produce secondary reactions at a local level of traumatic origin. Thus, local and regional hyperplasia of the oral mucosa is a frequently encountered effect in palatal expander cases [10]. This depends on the patient’s reactivity to mechanical damage and the distance of application of the expander to the mucosa, being favored by the presence of some local factors such as pre-existing carious lesions or tartar deposits [11]. Also, another cause of mucosal lesions in the case of orthodontic expanders is their overfrequent activation, a fact that does not allow the bone structures to adapt. In order to carry out the orthodontic treatment, the expander cannot be removed from the oral cavity, and as a consequence, treating mucosal hyperplasia becomes problematic.

The aim of this retrospective study was to evaluate clinical and histopathological changes that occur at gingival and palatal mucosa levels during palatal expansion treatment and their evolution during treatment. The null hypothesis of the present study was that palatal expanders do not cause mechanical trauma after insertion of this device and that there are no differences between post-therapeutic sites treated with conventional tooth-borne RPE techniques and mini-implant RPE.

## 2. Materials and Methods

### 2.1. Specimens and Sampling Technique

**Figure 1:**
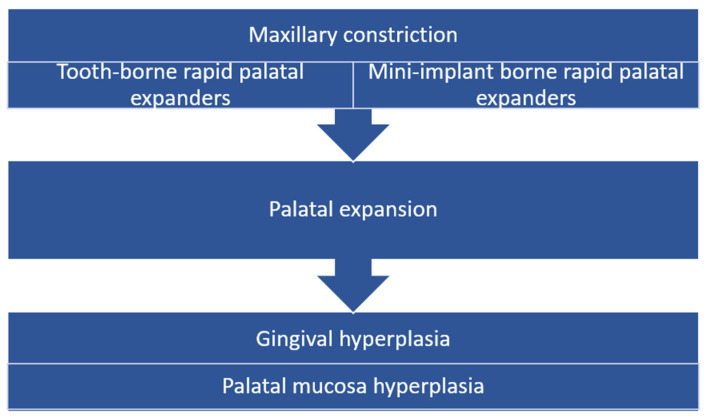
PICO chart followed within study protocol.

The authors chose the PICO framework [12] in order to achieve the objectives of this study, since it is the most commonly used model for structuring clinical questions (Figure 1). A power analysis was conducted to calculate the sample size. Based on previously available literature [6,8,9], using a package (Pwr) in R software (Bioconductor™, version 3.18 for Windows™, Boston, MA, USA) with a 95% confidence interval and 80% power of the study, the sample size was determined. This study was conducted in compliance with the Declaration of Helsinki. Ethical clearance was obtained from the Institutional Review Board of the Sc Algocalm Srl Private Medical Clinic, number 994/01.02.2023. Twenty-five (*n* = 25) research participants, of whom seventeen (*n =* 17) were females and eight (*n* = 8) were males, aged between thirteen and twenty-six years old, were examined for this retrospective study after written informed consent was obtained from them or their legal guardians. 

The inclusion criteria for this study were as follows:

- Gingival and palatal mucosa enlargement associated/consecutive with the rapid palatal expansion procedure.
- No medication for associated systemic conditions.
- The exclusion criteria for the study group were:
- Smokers.
- Patients with cardiovascular disease, diabetes, or epilepsy.
- Associated systemic medication, which could induce soft tissue modifications.

### 2.2. Palatal Expander Placement

**Figure 2:**
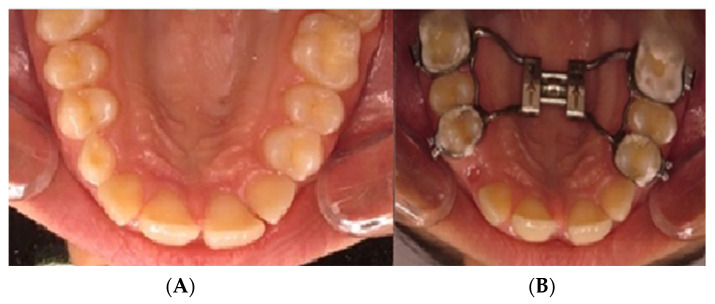
Images of the study group treated with tooth-borne appliances: (**A**) initial situation; (**B**) after RPE insertion.

After clinical and radiographical examination, the diagnosis of maxillary constriction was confirmed, and the decision for the rapid palatal expansion treatment method was taken. In the study group, sixteen (*n* = 16) patients were treated using tooth-borne palatal expanders (Figure 2). The expanders were inserted into the oral cavity of the patients at the level of the posterior third of the hard palate. The palatal expanders were placed 1–3 mm from the palatal mucosa at the time of insertion.

**Figure 3:**
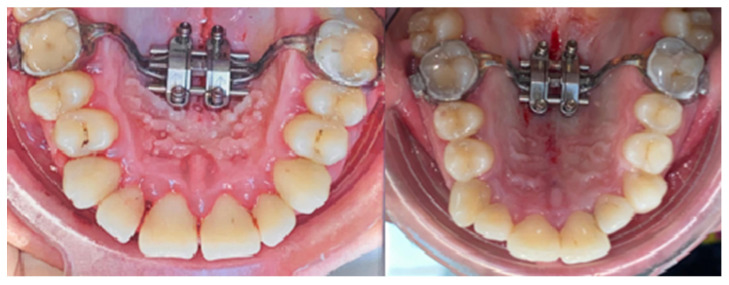
Hybrid anchorage in the study groups; four mini-implants supported RPE.

Nine (*n* = 9) research participants were treated using mini-implant anchorage rapid palatal expanders (MARPE) (Figure 2). The anchorage achieved in these cases was of a hybrid type, made on four mini-implants (Figure 3) inserted into the patient’s hard palate and bilaterally on the first molar and/or first premolar by means of metal rings.

### 2.3. Assessment of the Study Group

**Figure 4:**
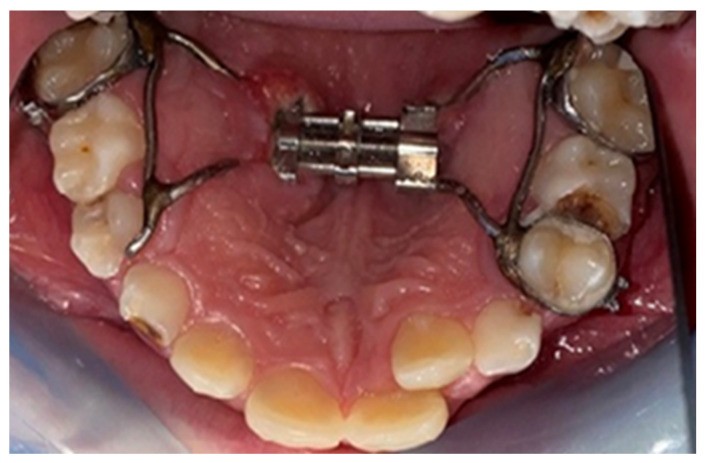
RPE expander inserted into the oral cavity. The presence of mucosal changes at the local-regional level and the presence of favorable factors (carious lesions) were observed.

**Figure 5:**
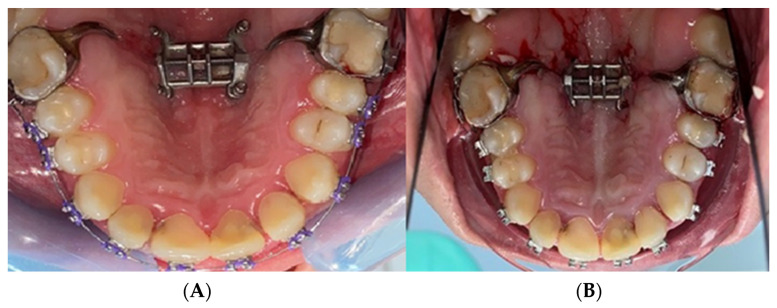
(**A**) MARPE expander with hybrid anchorage inserted into the oral cavity; (**B**) an accentuated vascular pattern was observed at the six-week check-up stage.

**Figure 6:**
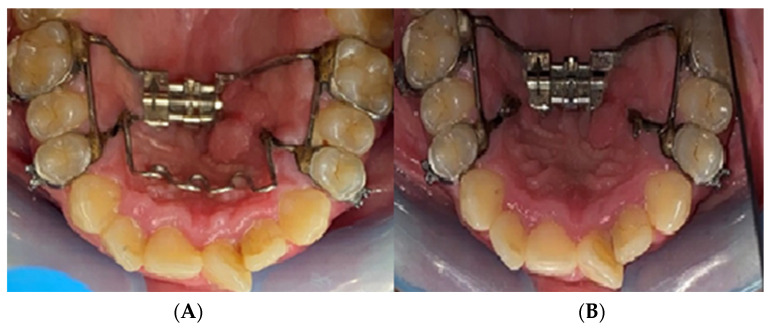
(**A**) RPE-type expander equipped with a tongue crib to combat infantile swallowing; (**B**) clinical aspect after the removal of the tongue crib.

**Figure 7:**
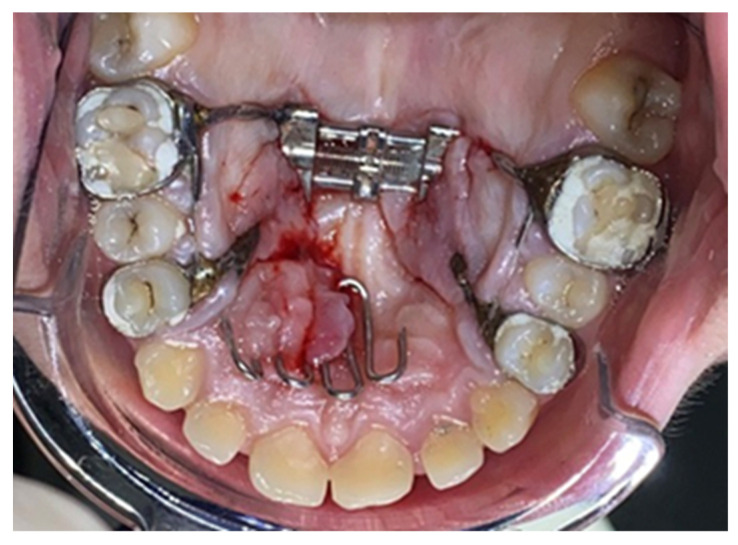
Severe inflammatory reaction of the palatal and gingival mucosa.

Following the application of the expander, the research participants presented themselves for regular checks (four- to six-week intervals) or unannounced, in case of emergencies, where the presence or absence of the local inflammatory reaction of the mucosa was observed (Figure 4, Figure 5, Figure 6 and Figure 7).

**Figure 8:**
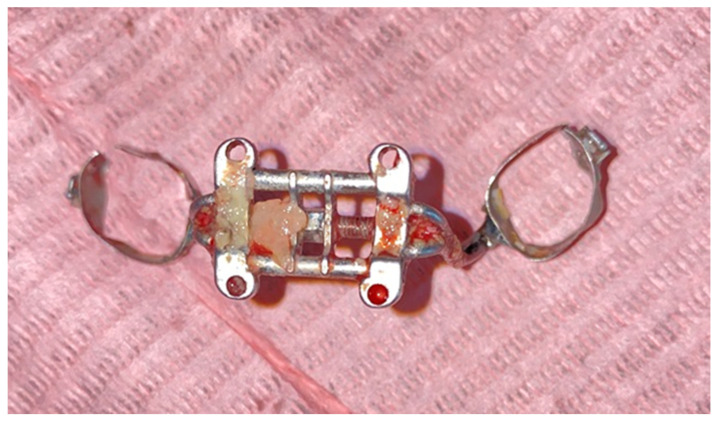
Expander removed from the oral cavity. Fragments of hyperplastic tissue were observed.

At the end of the treatment, following the removal of the expander (Figure 8), in order to examine the cellular changes within the hyperplasia of the palatal mucosa in palatal expander wearers and to confirm the diagnosis, fragments of tissue from the affected level were obtained via incisional biopsy and sent to the histopathology laboratory for a specialized examination. Surgical excision of the lesions was then performed, achieved under local anesthesia with the aid of topical application of lidocaine 2% (Septodont, Creteil, France), followed by intraoral infiltration of the hard palate using a solution of articaine and epinephrine 1:100,000 (ARTICAINE, Septodont), achieved with the aid of a syringe with a thin needle (0.30 × 38 mm). The biological product obtained was stored in a container with formalin and thus transported to the analysis laboratory in order to obtain a histopathological diagnosis.

### 2.4. Histopathological Analysis Protocol

The tissues sent for microscopic examination were fixed in formalin and embedded in paraffin. The paraffin blocks were cut with a microtome into 3 µm sections to conduct the Hematoxylin and Eosin (H&E) staining using the Thermo Scientific Gemini AS Automated Slide Stainer. Then, six sections were cut from the paraffin blocks for the immunohistochemical reactions. The primary antibodies used were anti-Pan keratin (AE1/AE3/PCK26) primary antibody and CONFIRM anti-Ki-67 (30-9). Rabbit monoclonal primary antibody, CONFIRM anti-CD3 (2GV6) rabbit monoclonal primary antibody, CONFIRM anti-CD20 (L26) primary antibody, CONFIRM anti-CD68 (KP-1) primary antibody, and CD31 (JC70) mouse monoclonal antibody were also used. All the immunohistochemistry studies were performed using a DAB detection kit on a Ventana BenchMark GX automated slide staining system. The slides were examined under a Carl Zeiss microscope (Carl Zeiss Microimaging GmbH, Jena, Germany) equipped with an AxioCam MRc5 color digital camera. The interpretation was performed by an experienced pathologist.

## 3. Results

**Figure 9:**
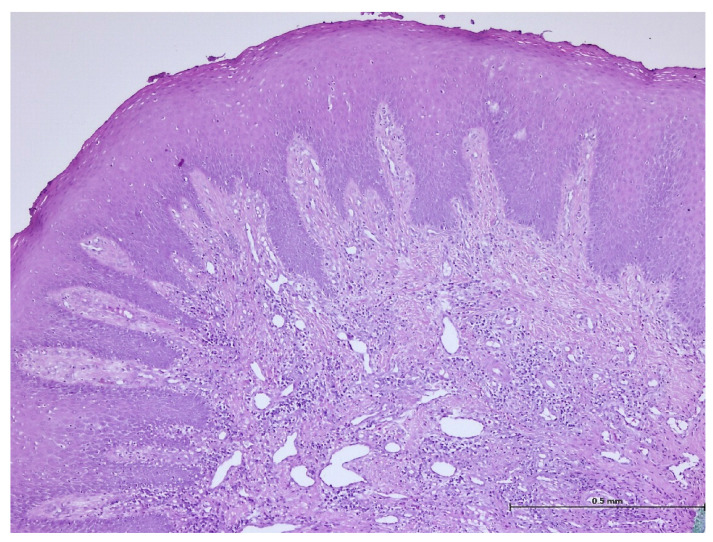
Histologic aspects in H&E staining (magnification ×100); marked acanthosis of the spinous layer was observed in the areas with a pseudo-epitheliomatous appearance.

**Figure 10:**
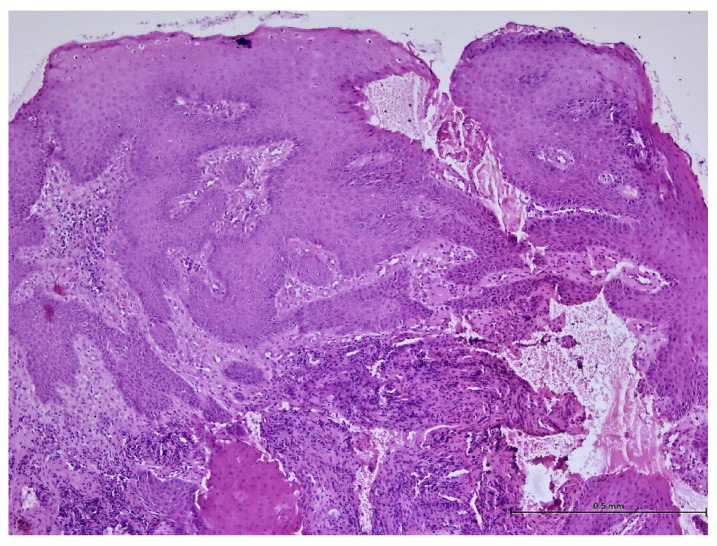
Histologic aspects in H&E staining (magnification ×100); the subepithelial connective tissue showed a moderate, polymorphic inflammatory infiltrate consisting of lymphocytes, histiocytes, plasma cells, and eosinophils.

**Figure 11:**
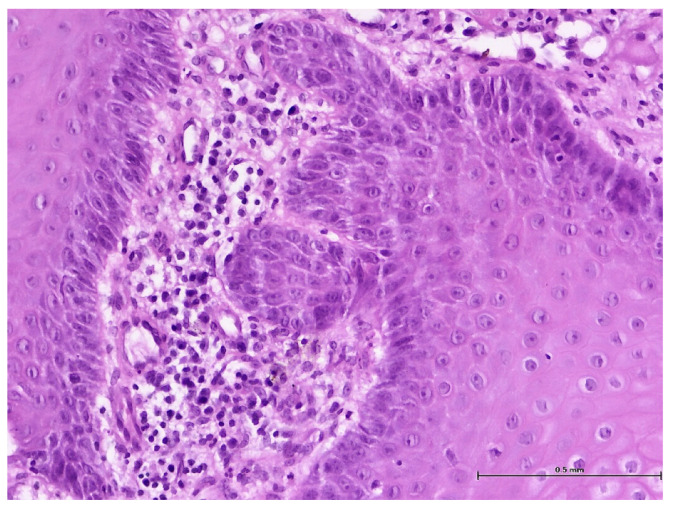
Histologic aspects in H&E staining (magnification ×200); interstitial edema, polymorphic inflammatory infiltrate, numerous small vessels with slightly ballooned endothelium, and red blood cells in the lumen.

**Figure 12:**
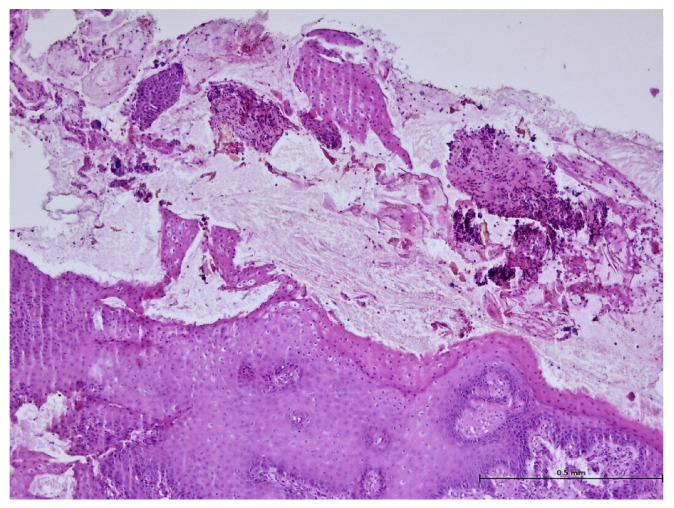
Histologic aspects in H&E staining (magnification ×100); dental plaque deposits were observed.

During the histopathological examination (Figure 9, Figure 10, Figure 11 and Figure 12), all cases (*n.* 25) presented the following characteristics:

The palatal mucosa was partially covered by keratinized stratified squamous epithelium with foci of parakeratosis. Marked acanthosis of the spinous layer was observed in the areas with a pseudo-epitheliomatous appearance. Acanthosis with elongation of ridges in the spinous layer was also observed. In the connective tissue of the subepithelial layer, there was edema, a small polymorphic inflammatory infiltrate consisting of rare lymphocytes, rare histiocytes, plasma cells, and eosinophilic granulocytes. Numerous small, thin-walled vessels, some with ballooned endothelium and dilated endothelium, with or without the presence of haematocytes, were also visible, along with dilated lymphatic vessels. In the middle of the fragments, marked acanthosis areas showed a keratinization tendency. In eleven cases (*n* = 11) on the epithelium’s surface, mucus deposits, fibrin-hematic material, and bacterial colonies (bacterial plaque) were observed.

**Figure 13:**
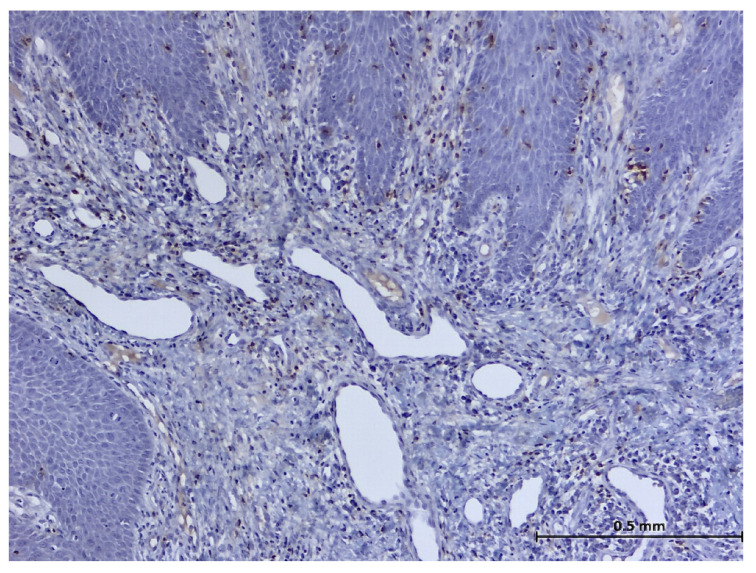
Immunohistochemistry: CD3 reaction highlights rare T lymphocytes (magnification ×100).

**Figure 14:**
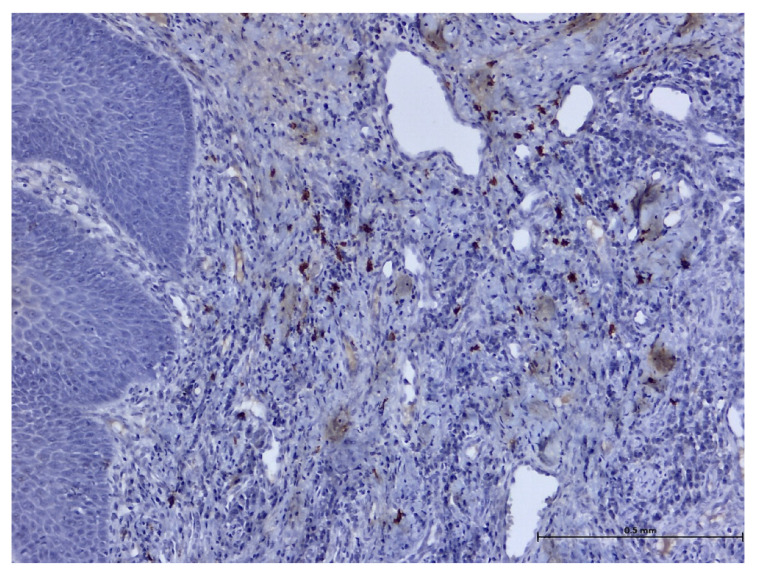
Immunohistochemistry: CD20 reaction highlights rare B lymphocytes (magnification ×100).

**Figure 15:**
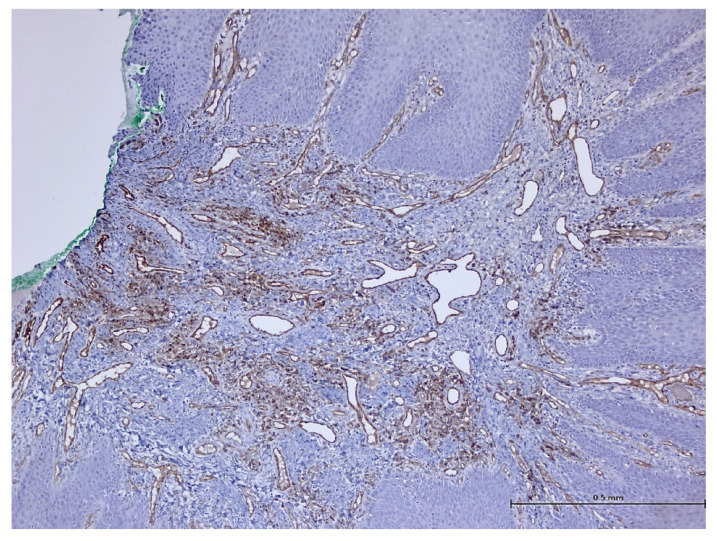
Immunohistochemistry: CD31 reaction marks endothelial cells of blood and lymphatic vessels (magnification ×100).

**Figure 16:**
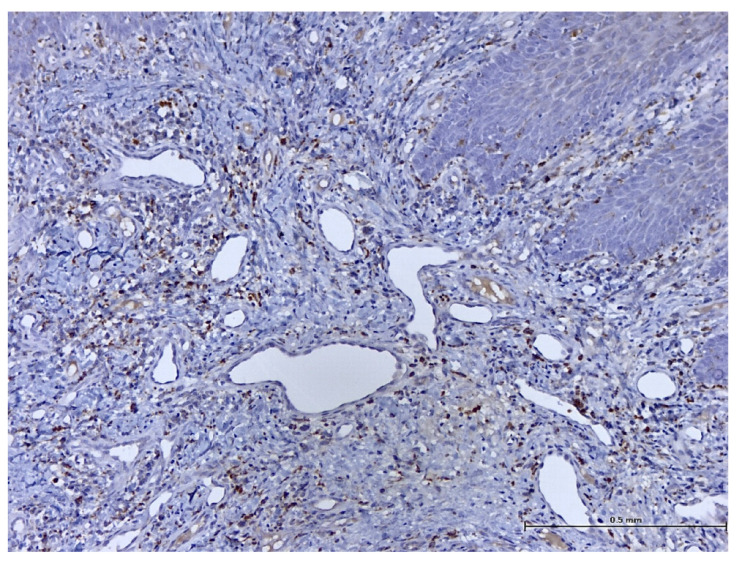
Immunohistochemistry: CD8 reaction shows a moderate number of macrophages (magnification ×100).

**Figure 17:**
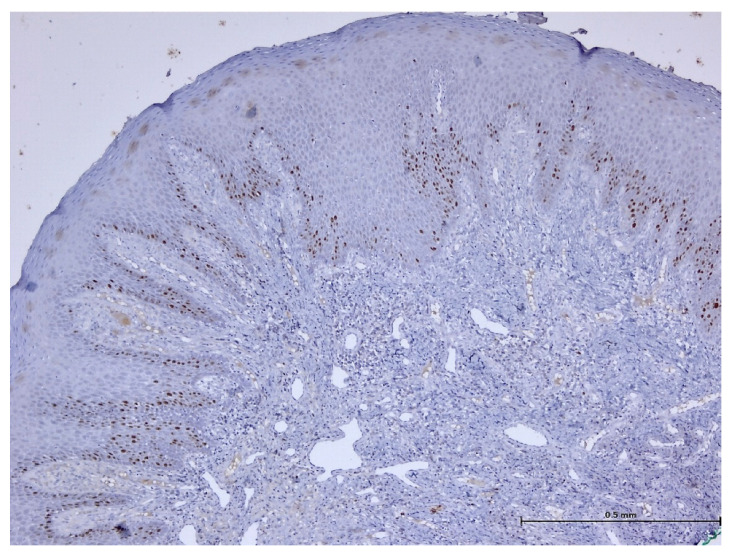
Immunohistochemistry: Ki-67 nuclear proliferation index marks the basal keratinocytes (magnification ×50).

**Figure 18:**
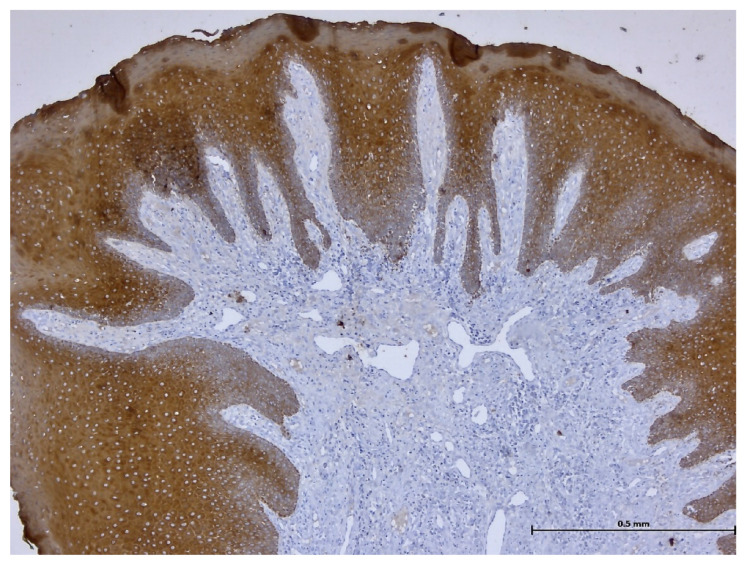
Immunohistochemistry: Anti-Pan Keratin marks the surface epithelium (magnification ×50).

After performing immunohistochemical reactions, the final histopathological diagnosis was reactive acanthosis of the palatal epithelium, likely due to mechanical causes, with a tendency for keratinization (Figure 13, Figure 14, Figure 15, Figure 16, Figure 17 and Figure 18).

The changes identified were specific to mechanical traumatic injuries, thus excluding hyperplasia from other etiologies (infectious, tumoral, or non-mechanical traumatic). The examined fragments showed hyperplasia, with widening of the spinous layer (acanthosis) without acantholysis and without hyperkeratosis. The elongated ridges showed a slight tendency to merge. The epithelium was mostly destroyed and partially replaced by granular tissue. Around the large vessels, with their partial interest, numerous granulocytes were highlighted, penetrating from the outside towards the middle of the vessel. Histologically, the masticatory mucosa has a partially ortho-keratinized and para-keratinized stratified squamous epithelium. The amount of keratinization of the oral mucosa reflects the amount of stress or mechanical abrasion that the region experiences. An ortho-keratinized epithelium contains keratinocytes with keratin and nuclei, whereas the para-keratinized epithelium lacks nuclei. Differentiating between ortho- and para-keratinized tissue is based on appearance and has no clinical significance. Because this mucosa is generally under higher stress levels, it has more pronounced dermal papillae and rete ridges than the oral lining mucosa. The layer of connective tissue termed lamina propria is located underneath the epithelium and comprises collagen, blood vessels, neurons, fibroblasts, and a small number of inflammatory cells.

Trauma caused by the insertion of an expander into the oral cavity was considered to be the causative factor of the inflammatory reaction. The continuous irritating factor was represented by the presence of the expander, and, in some cases, the tongue crib was considered a contributing factor to the inflammation.

## 4. Discussion

This study was performed in order to improve our insight into the role of palatal expanders that might induce palatal and gingival overgrowth during orthodontic force application. On average, after six months of expander placement, the authors detected, using clinical and histopathological examinations, a clear diagnosis of mechanically induced hyperplasia. From a total of twenty-five (*n*. 25) cases analyzed, sixteen patients were treated with tooth-borne rapid palatal expanders, and nine (*n*. 9) were treated with mini-implant-assisted rapid palatal expansion devices. The degree of severity varied from one patient to another, especially depending on the distance from the palatal mucosa where the expander was inserted, the reactivity of the patient, compliance with the recommendations related to the expander activation, and the hygiene of the patients. These results are consistent with similar previous studies published in the literature [13,14,15,16]. Furthermore, as suggested by previous studies [17,18,19], although the occurrence of hyperplasia was not directly correlated with the patients’ hygiene, patients with inadequate hygiene presented a high degree of severity of inflammation. The presence of bonded fixed orthodontic appliances also had a negative impact on gingival health.

Of the twenty-five cases, the insertion distance between the palatal mucosa and the appliance was less than 2 mm in five patients, 2 mm in twelve patients, and 3 mm in eight patients. On average, patients with an expander inserted at 3 mm presented a lower degree of inflammation of the palatal mucosa without influencing the results, followed by orthodontic treatment. Patients with the expander at a distance of 1.5 mm presented severe mucosal hyperplasia. Patients with an expander inserted at 2 mm showed a variable degree of hyperplasia. Considering these aspects, the authors conclude that the proximity of the expander to the mucosa represents an irritating thorn which has a role as a favoring factor in the occurrence of inflammation. Among the twenty-five cases, only two patients did not fully comply with the indications related to expander activation; overactivation was observed in those cases. In these cases, patients presented a higher degree of hyperplasia, confirming the negative role of overactivation due to the non-adaptation of bone structures to the new clinical situation. These results were also observed by previous researchers like Jeon et al. (2002) [20] and Yacout et al. (2022) [21].

Six of the patients described an increased sensitivity to mechanical trauma, with the rapid appearance of ecchymoses or hematomas even at low intensities of the traumatic factor. However, no significant differences were observed between these patients and the rest in terms of the severity of the inflammatory reaction in the oral mucosa. Schuster et al. [22] reported medical complications such as pain and decubitae in a third of the inquired offices, but they suggested that side effects of RME are often temporary and permanent damages are rather rare. No significant differences were observed in the degree of hyperplasia in male versus female patients. Related to the age of the patients, the study group between thirteen and sixteen years old showed, in general, more severe forms of hyperplasia compared to patients over sixteen years old. Further studies conducted by Bishara et al. [23] regarding the age of patients concluded that the side effects of RME tend to be smaller in children than they are in adolescence and adults and are associated with the degree of skeletal maturity. Furthermore, Capelozza et al. [24] noticed multiple side effects, such as pain, edema, and ulceration, when using palatal expansion in adult patients. In the present study, although the reactions were more severe, no biological samples were collected to see the blood level of hormones, but the authors suspect this factor to be relevant, considering the results obtained in the mentioned age categories. Future studies can be relevant to support these findings and to observe if there could be a possible relationship between blood hormone levels and the degree of inflammation. As a treatment option for persistent palatal hypertrophy associated with maxillary expansion procedures, Omezli et al. [13] suggest that, under general anesthesia, the hypertrophic areas in the palatal region be excised with the help of a scalpel and electrocautery to smooth the area of the palatal mucosa in order to prevent food retention.

Careful design and application of the MARPE appliance can achieve successful transverse expansion of the maxilla and the surrounding structures in patients beyond the age typically considered acceptable for traditional rapid palatal expansion, with minor buccal tipping but without bone loss or trauma of the palatal mucosa [25]. Tsai et al. [26] conducted a study on twenty-nine patients treated with MARPE and found that 48.3% of the subjects reported swelling or inflammation over the palatal mucosa, 41.4% of the subjects complained of difficulty cleaning around the device, and 37.9% experienced soft tissue impingement during expansion.

The widening of maxillofacial spaces is a significant factor in the improvement of nasal breathing, a major advantage of the use of RPE. In children, the use of RME can reduce tonsillar and adenoidal volume, as reported by Yoon et al. [27]. Some of the most significant results of the use of RPE in the aforementioned study are the reduction in adenoidal volume experienced by 90% of children and the tonsillar volume reduction experienced by 97.5% of children. The average volume decreases were 20.1% and 40.2%, as measured by CBTC. RPE usage shows other significant positive effects, including a reduction in oral breathing, likely associated with enhanced nasal philtrum [28]. This also led to a lower rate of nasal-respiratory infections.

The use of RPE can also have positive implications for reducing the effects of obstructive sleep apnea (OSA) [29]. This is likely due to a combination of factors, including lower tongue collapse incidence associated with persistent oral breathing, adeno-tonsillar volume decreases, and pharyngeal segment stiffening. Another positive impact of the use of RPE is the improvement of Eustachian tube function. It improves the strength of the tensor palatine muscles and the function of the elevator, leading to a reduced occurrence of ear infections, a leading cause of hearing loss. 

Previous researchers, like Stasiak et al. [30], demonstrated in a very comprehensive systematic review that it is not possible to establish the generally recommended target site for the placement of the mini-implants in the area. The high variability of bone measurements and the lack of reliable predictors of bone availability justify the use of CBCT for mini-implant trajectory planning. 

Although no significant results were found in this study regarding a possible correlation between gingival overgrowth and the age of the patients, some authors, like Guan et al. [31], emphasize the importance of early orthodontic treatment as it helps patients achieve balanced development of masticatory function.

The reduction in bone density through the removal of myeloid HIF1 and impaired Acp5 and Rank1 gene expressions leads to significantly higher induced tooth movement. This has been demonstrated in recent experiments by Kirschneck et al. [32]. It could be an alternative therapeutic approach to lower treatment-related periodontal hazards.

The strengths of this study reside in the histological examination, which revealed the mechanical character of the lesion, strengthening the causal relationship between the insertion of the expander and the occurrence of hyperplasia. This is based on histological indicators, including acanthosis phenomena and the destruction of epithelial tissue, followed by its replacement with granulation tissue. 

This study had several limitations. The suggested methodology is difficult to apply in practice considering the costs of histopathological examination. Moreover, the study needs to be applied to an adequate number of cases to confirm whether the type of design and activation time could influence the results and to follow both the clinical and stability outcomes of the use of palatal expanders. Comparative studies can be conducted to evaluate the efficacy of these devices. Long-term studies assessing this clinical situation could provide valuable insights. Clearly, a more thorough analysis is needed to fully understand the cause of malocclusion and define proper treatment.

## 5. Conclusions

Following the results of this study, the authors can conclude that the type of palatal expander influences the degree of inflammation, the severity of hyperplasia was more pronounced in the case of MARPE than in the case of RPE usage, the analysis of the distance between the expander and the palatal mucosa did not provide conclusive results, and the incidence and severity of the reaction were variable in patients with the same distance between the expander and the palatal or gingival mucosa. Additionally, overactivation of the expander caused increased hyperplasia of the palatal mucosa. The histopathological examination revealed the mechanical character of the lesion, strengthening the causal relationship between the insertion of the expander and the occurrence of hyperplasia of the palatal mucosa.

## Data Availability

The supporting information for this research can be checked with the corresponding authors at: alexandru.vlasa@umfst.ro; anamaria.bud@umfst.ro.

## List of Abbreviation

RPE	Rapid Palatal expander
MARPE	Mini-implant anchorage rapid palatal expander
PICO	Population, intervention, control, and outcomes
Pwr	Power
H&E	Hematoxylin and Eosin
AE1/AE3/PCK26	Anti-Pan Keratin primary antibody
CONFIRM Anti-Ki-67	Rabbitt Monoclonal Primary Antibody
CD31 (JC70)	Mouse Monoclonal Antibody
